# Additional Value of SPECT/CT to Tc-99m MAG3 Renal Scintigraphy in the Diagnosis of a Patient with Ureteroileal Fistula

**DOI:** 10.4274/Mirt.44

**Published:** 2012-08-01

**Authors:** Nilüfer Yıldırım Poyraz, Elif Özdemir, Mutlay Keskin, Şeyda Türkölmez

**Affiliations:** 1 Ankara Atatürk Training and Research Hospital, Department of Nuclear Medicine Ankara, Turkey

**Keywords:** Urinary fistula, radioisotope renography, single-photon emission computerized tomography, X ray computerized tomography

## Abstract

We performed Tc-99m MAG3 dynamic renal scintigraphy followed by a SPECT/CT imaging to a 38-yr-old woman who had a fistula in the lower urinary tract after a gynecological operation for diagnostic purposes. After scintigraphy, CT and fusion images were evaluated, it was observed that the activity in the right lower quadrant was actually in the ileal lumen. When early and late SPECT/CT images were compared, it was seen that the activity was moving distally with intestinal peristaltism. The reason for diagnostic imaging of the fistulas is not only to show the existence of the fistula but also locate it anatomically before the surgery. SPECT systems integrated with CT scanning provide functional and anatomical imaging at the same session. Dynamic renal scintigraphy and abdominal SPECT/CT, is a safe diagnostic procedure in visualization of urinary tract fistulas with advantages of the low cost, low radiation exposure and easier tolerability compared to double contrast imaging.

**Conflict of interest:**None declared.

## INTRODUCTION

Urinary system fistula is a rare condition which is seen mostly as a complication of surgery/radiotheraphy and trauma. It may also happen spontaneously. The two most commonly seen types of fistulas are ureterovaginal and ureteroenteric fistulas, usually encountered as a surgery complication. Nearly 75% are seen after abdominal or vaginal hysterectomy operation (1). Fistulas are seen in about 2.5% of patients with gynecological malignancies as a complication of surgery, and they can be diagnosed years after the therapy. Child delivery traumas, urological interventional procedures and pelvic irradiation are among the other frequently seen causes. 

Symptoms may differ according to the type of the fistula, but abdominal pain with recurrent urinary tract infections are the common ones. Therapy is the surgical correction for fistulas (2). When there is suspicion of a fistula, first thing to do is to show the anatomical localization of the urinary leakage. Excretory urography, dynamic renal scintigraphy (DRS) and computerized tomography (CT) are the noninvasive imaging procedures for this purpose (3,4). 

DRS is extremely sensitive in evaluating renal function and urinary leakage. But it should be combined with three dimensional imaging techniques to view the localization as it is a planar imaging method. Gamma camera systems integrated with CT gives us the opportunity to have functional and anatomical imaging at the same session. In this case, we have evaluated the patient suspected of having a fistula between urinary and gastrointestinal tracts with DRS followed by a SPECT/CT. 

## CASE REPORT

A 38-yr-old woman who had a history of cervical carcinoma, was admitted to our hospital with the complaints of abdominal pain, dysuria and hematemesis. She had a total abdominal hysterectomy and bilateral salpingo-ooforectomy (TAH-BSO) operation two years ago and during the operation it was seen that right ureter was lacerated and an ureterovesical catheter was placed after it was mended with ureterourethral anastomosis. In the ultrasonographic follow-up, a pelvicalyceal dilatation was seen in the right kidney and the renal function tests were found to be normal. The patient had recurrent urinary tract infections for the previous 6 months and she also has hematuria and hematochezia. After the upper gastrointestinal system endoscopy and colonoscopy, no specific findings were seen. E. coli was isolated in urinary culture. Then the patient was sent to our clinic for further evaluation of the kidney functions and probable ureteral abnormalities. 

5 mCi Tc- 99m labeled MAG-3 was administered intravenously to the patient and planar dynamic imaging was performed in posterior projection. (LEHR collimator, with 128x128 matrix, 140±20 % keV) 60 images each of 1 sec and 60 images each of 30 sec were obtained. Images were evaluated visually and quantitatively. 

**Figure 1:**
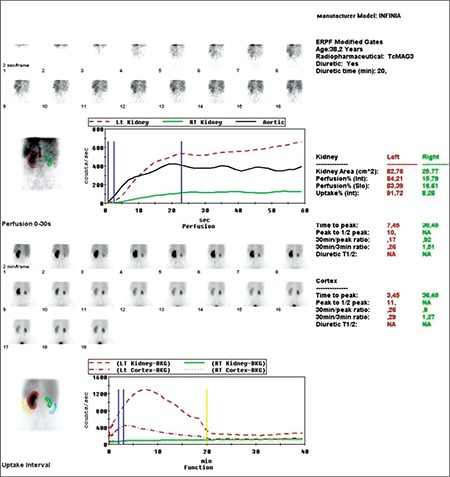
Dynamic renal scintigraphy and renogram curves

**Figure 2:**
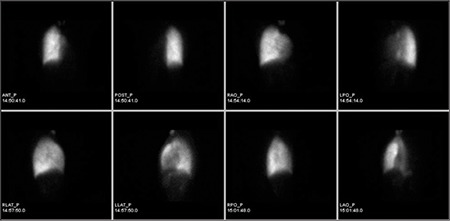
Anterior and posterior static images show the mild activity in the right lower quadrant

**Figure 3:**
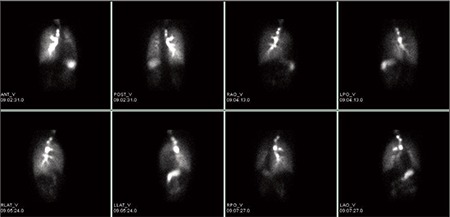
The plane fused SPECT/CT images of the abdomen show the activity in the ileal lumen

**Figure 4:**
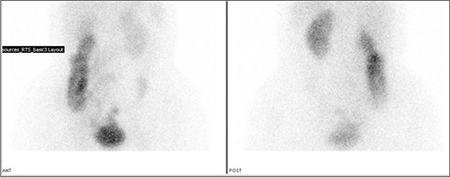
Anterior and posterior static images (late phase)show the activity localization which is changed to distally

**Figure 5:**
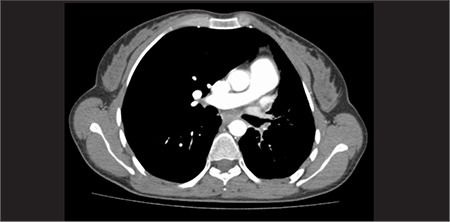
The plane fused SPECT/CT images of the abdomen show the activity in the ileal lumen which is changed to distally

Left renal function was found to be normal and the contribution to the total renal function was calculated to be 87%. But, right kidney was found to be smaller in size, perfusion and concentration functions were lower. In the excretion phase, a pelvicalyceal stasis were observed without diuretic response in the right kidney (Figure 1). During dynamic imaging nonhomogenous activity in the right lower quadrant was seen and that activity was increased with time. Right after that, static imaging (Figure 2) and SPECT/CT imaging were performed to the same localization. SPECT/CT images were obtained using two-headed gamma camera integrated with low energy X-ray system (Infinia-Hawkeye, GE). Transaxial, coronal and sagittal images were combined to have three dimensional images for abdomen. After scintigraphy, CT and fusion images were evaluated, it was observed that the activity in the right lower quadrant was actually in the ileal lumen (Figure 3). Static images (Figure 4) and SPECT/CT (Figure 5) were repeated after 20 minutes. When early and late SPECT/CT images were compared, it was seen that the localization of activity was changed to distally with intestinal peristaltism (Figure 6 a, b). 

## LITERATURE REVIEW AND DISCUSSION

Ureteroenteric fistulas are abnormal connections between ureters and intestines. They are usually seen as ureterocolic type, and ureteroileal ones are less frequent. Pelvic surgery, colorectal malignancies and radiotherapy are common etiological factors (5). Patients usually have abdominal pain, hematuria, recurrent urinary tract infections, pneumoturia, fecaluria and diarrhea. Double contrast barium enema is the most frequently used diagnostic procedure (4) but it needs to be confirmed with a cross sectional anatomical imaging technique (3). As the patient comfort and radiation exposure are considered, newer techniques are needed to replace barium enema. 

DRS is a noninvasive and sensitive imaging technique for evaluating the renal function and displaying the urinary leakage. SPECT is widely used in nuclear imaging techniques for its advantage of having a three dimensional image. SPECT systems integrated with CT, provides functional and anatomical imaging at the same session. It is easier to locate the fistula with SPECT/CT after DRS, and no extra radioactivity administration is needed. DRS and abdominal SPECT/CT, is a safe diagnostic procedure in visualization of urinary tract fistulas with advantages of the low cost, low radiation exposure and easier patient tolerability compared to barium enema examination.

## Figures and Tables

**Figure 6a:**
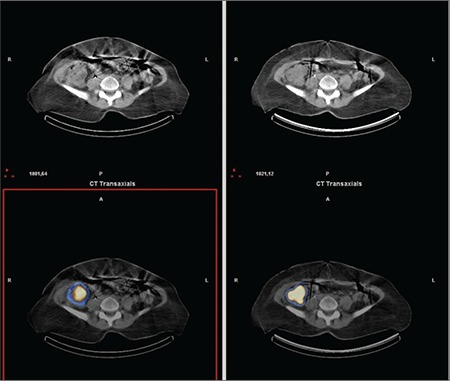
Comparison of early and late phase SPECT/CT images; transaxial slices

**Figure 6b:**
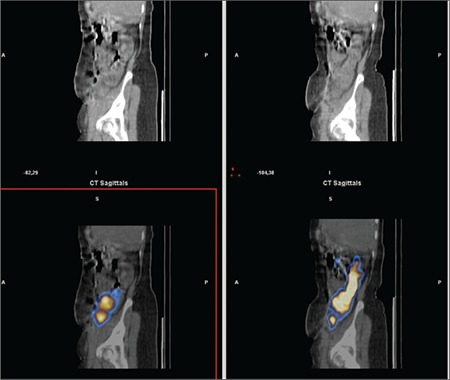
Comparison of early and late phase SPECT/CT images; saggital Slices
